# Around the Hospitals

**Published:** 1894-05-19

**Authors:** 


### Around the Hospitals.

[Notice to the Managers of Institutions.—Special space is now reserved for the insertion of notices of hospital meetings and festivals. The Editor requests that all notices may reach The Hospital Office, 428, Strand, at least one week before the date of each meeting.]

**The Wrexham Infirmary.**—The sixty-first annual report of this infirmary shows that good progress has been made during the past year. Structurally, the improvements have consisted in the conversion of the old laundry and a room adjoining into an out-patients' waiting-room, dispensary, and consulting-room, whilst part of the fever hospital has been made into quarters for the private nursing staff. A balcony is nearly finished, which will add to the comforts of patients in the Jubilee ward, and serve as an extra escape in case of fire. It is with regret that we learn that the very small sum of £300 required to carry out the improvements has not yet been subscribed. To add to the completeness of the provision for patients, steps are being taken to secure a Welsh-speaking nurse. For some time past a working man has acted as visitor to the infirmary, elected by the North Wales Miners' Federation eyery month, and this has been found a satisfactory step in every way. At the annual meeting it was proposed and carried that a working man's representative should be added to the committee. Financially, the infirmary is not in a strong position, and it is hoped that stimulated interest will cause an increase of funds. During the past year 198 in-patients and 838 out-patients were treated.

**The Royal Hospital for Diseases of the Chest.**—This hospital would seem to have entered on a new lease of prosperity. It has been enabled to open and furnish two new wards. The "Albert Edward" and "Shaftesbury" bring up the total available beds to 80. Although the careful economy exercised shows that expenses are curtailed as far as possible, additional beds means additional expenditure, and the income of the hospital is not yet adequate for its wants. The successful annual dinner of last year resulted in a good collection, and in a number of new annual subscriptions, and we hope these will be the forerunners of a still larger amount. The list of presents for the patients shows much thoughtful kindness. Her Majesty the Queen, bearing in view the special diet required by the sufferers, contributed no less than 175 bottles of wine, whilst Messrs. Burroughs and Welcome sent a useful gift of cod liver oil, and numerous other friends contributed to the welfare of the patients. During the year 465 in-patients and 8,972 out-patients were treated. The ordinary income amounted to £6,752, and extraordinary to £1,110, making a total of £7,862. The expenditure was £7,321, in which is included the sum applied to the furnishing and opening of the new wards, together with repairs and cleaning.

